# The clinical phenotypes and therapeutic strategies for stiff skin syndrome: a case series with literature review

**DOI:** 10.1186/s13023-025-03748-7

**Published:** 2025-06-23

**Authors:** Caihui Zhang, Sihao Gao, Zhixing Sun, Tao Wang, Hongmei Song

**Affiliations:** 1 Department of Pediatrics, Peking Union Medical College Hospital, Peking Union Medical College, Chinese Academy of Medical Sciences, Beijing, China; 2 Department of Dermatology, Peking Union Medical College Hospital, Peking Union Medical College, Chinese Academy of Medical Sciences, Beijing, China

**Keywords:** Stiff skin syndrome, Children, Diagnosis, Treatment, Prognosis

## Abstract

**Background:**

Stiff skin syndrome (SSS) is a rare, non-inflammatory skin disease with a pronounced restriction in joint mobility. In this study, we aim to report Chinese pediatric patients with SSS in our center and summarize the clinical features of the disease through literature review.

**Results:**

A retrospective study was conducted on 16 pediatric patients diagnosed with SSS at Peking Union Medical College Hospital between January 2014 and January 2024, based on clinical manifestations, laboratory tests, and skin biopsy findings. Among these cases, two were classified as widespread SSS, and 14 as segmental SSS. Additionally, a review of relevant literature published between January 2000 and January 2024 involving 138 cases of pediatric SSS was also conducted. The clinical characteristics, treatment, and prognosis of these 154 patients were summarized. The age of onset in patients was 2.0(0.5, 4.8) years, with an average age at diagnosis being 9.0(5.0, 13.0) years. Thigh skin sclerosis (81, 52.6%) was the most common manifestation observed in these patients. Joint restriction was present in 55(35.7%) patients. Patients with joint contractures had longer diagnostic delays compared with those without joint contractures. Patients were primarily treated with physical therapy, while some patients received medications such as mycophenolate mofetil (MMF), losartan, and secukinumab. However, the prognosis varied.

**Conclusion:**

The diagnosis of SSS should involve a thorough investigation of family history, detailed physical examination, comprehensive pathological assessment, genetic testing when applicable, and careful exclusion of other scleroderma-like diseases. Currently, there is limited evidence supporting the use of systemic treatment options targeting the transforming growth factor-β or interleukin-17 pathways (such as MMF, losartan, and secukinumab) to slow disease progression. However, these treatments are not capable of reversing established skin lesions, and further investigations are imperative to assess their therapeutic efficacy in SSS.

## Background

Stiff skin syndrome (SSS) is a rare autosomal dominant genetic disorder that was first reported by Esterly and McKusick in 1971 [1]. SSS shares some similarities with scleroderma and is characterized by skin thickening and sclerosis, that can lead to restricted joint mobility and contractures, in the absence of visceral, musculoskeletal, vascular, or immunologic abnormalities. Hyperpigmentation and hypertrichosis can also develop in affected areas [2]. The syndrome can be divided into segmental and widespread subtypes according to the distribution of the lesions, age of onset and functional impairment [3]. Given the rarity of this disease and the similarity of its cutaneous symptoms to those of scleroderma and eosinophilic fasciitis, a significant proportion of patients are misdiagnosed during their initial consultations with pediatric rheumatologists, leading to unnecessary immunosuppressive therapies that could significantly compromise their quality of life. The treatment is challenging and there are no established guidelines for patient care [4]. Therefore, this study is to retrospectively analyze the clinical characteristics, diagnosis, treatment and prognosis of SSS patients to provide valuable insights for clinical management. Concurrently, we analyzed the high-risk factors contributing to joint contractures, aiming to facilitate early intervention and improve outcomes.

## Methods

### Patients

We conducted a retrospective observational study of 16 consecutive cases of childhood-onset SSS diagnosed based on clinical and histopathological features between January 2014 and January 2024 at Peking Union Medical College Hospital (PUMCH). The patients were followed for at least 1 year since diagnosis. Those without clinical data and/or histopathological slides were excluded from the study. Therapeutic efficacy was defined as both softening of the skin at original sclerotic sites and the absence of new sclerotic lesions. This research was performed under the guide of the Declaration of Helsinki and approved by the Institutional Review Board of Peking Union Medical College Hospital (PUMCH) (JS-3362D).

### Literature search

We searched the literature in PubMed, Wanfang data, and China National Knowledge Infrastructure (CNKI) database for relevant studies of childhood-onset SSS patients published between January 2000 and January 2024 using the keyword “stiff skin syndrome”. Cases with age of onset greater than 18 years old were excluded. We also removed duplicate cases in different studies. Cases with detailed clinical data were collected for further analysis of their demographic information, clinical manifestations, pathological findings and the response to treatment.

**Fig. 1:**
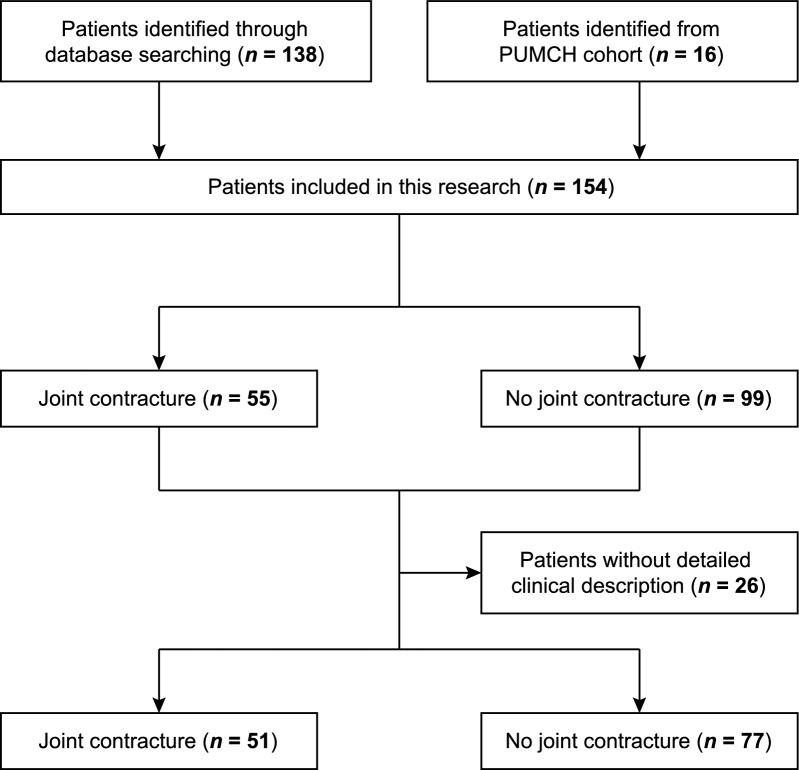
Flow diagram of the study

We retrospectively analyzed clinical data from 138 SSS patients reported in the literature and 16 SSS patients from PUMCH cohort to further investigate their demographic characteristics, clinical presentations, pathological findings, and treatment responses. Fifty-one patients with detailed joint symptoms were included to summarize their joint involvement characteristics (Fig. 1).

### Statistical analysis

For descriptive statistics, median and range were used for numerical data that may not follow a normal distribution. The arithmetic mean and standard deviation were calculated for normally distributed numerical data. Categorical data were described with frequencies and proportions. The notation *n/N* represents the number of positive cases (*n*) over the total number of examined cases (*N*). Chi-square test or non-parametric test was used for comparing prevalence of clinical manifestations between patients with or without joint contractures. Statistical analysis was performed using SPSS software (Version 26.0) and GraphPad Prism (Version 9.3.1).

## Results

### Clinical findings

Sixteen patients (8 males and 8 females) who visited PUMCH during the period of January 2014 to January 2024 were diagnosed with SSS (Table 1). Fourteen cases were segmental SSS and only 2 cases were widespread SSS, with the latter being exclusively female. The age of onset for 16 patients was 4.0 ± 2.0 years, with a mean age at diagnosis of 8.4 ± 4.5 years, and an interval between disease onset and diagnosis being 4.4 ± 3.4 years. Although two patients had a family history of scleroderma in their fathers and two in their mothers, genetic testing did not reveal pathogenic variants. Some patients were initially misdiagnosed with scleroderma (6 patients), telangiectasia (1 patient), or connective tissue nevus (1 patient). These patients underwent skin biopsy after ineffective treatments, ultimately confirming the diagnosis of SSS.

**Table 1:** Clinical features of 16 patients with stiff skin syndrome followed at our hospital

Case No	Sex/onset age	Clinical manifestations			Autoantibodies	Skin biopsy				Treatment	Follow-up
		Distribution	Overlying skin symptoms^a^	Joint limitation		Fibroblast proliferation	Dermal thickening	Adipocyte infiltration	Mucopolysaccharide deposition		
Widespread SSS											
1	F/4.0	Left lower limb, lumbar area and right upper extremities	Subcutaneous nodules	Yes	Negative	Yes	Yes	No	**–**	Chinese patent medicine	Stable
2	F/1.0	Left thigh, upper extremities	Subcutaneous nodules	No	anti-proliferative nuclear antigen antibody	Yes	Yes	**–**	Yes	Chinese patent medicine	Stable
Segmental SSS											
3	M/7.0	Right thigh, lumbosacral region	Hyperpigmentation, hypertrichosis	No	Negative	Yes	Yes	Yes	**–**	Phototherapy, MTX, MMF	Stable
4	F/5.6	Left thigh	Hyperpigmentation, hypertrichosis	No	Negative	Yes	Yes	Yes	**–**	Phototherapy, Chinese patent medicine, MMF	Stable
5	M/2.0	Left buttock, left thigh	None	No	Negative	Yes	Yes	Yes	**–**	**–**	Stable
6	M/4.0	Left lower limb	None	Yes	Negative	Yes	Yes	Yes	**–**	Phototherapy, tofacitinib	The lesion became larger and harder
7	F/1.0	Right shoulder, right upper limb to right wrist	Hypertrichosis, subcutaneous nodules	No	anti-ribonucleoprotein antibody	Yes	Yes	**–**	Yes	Baricitinib	Non-responsive
8	F/2.5	Left hip, left buttock, left thigh	Subcutaneous nodules	Yes	Negative	Yes	Yes	Yes	**–**	Chinese patent medicine	Stable
9	M/3.8	Left thigh	Hypertrichosis	No	Negative	Yes	Yes	**–**	Yes	Chinese patent medicine	Lost to follow-up
10	F/2.0	Forehead, right lower back	None	No	Negative	Yes	Yes	Yes	**–**	Chinese patent medicine	Stable
11	M/4.0	Left lower limb	None	No	Negative	Yes	Yes	Yes	Yes	**–**	Stable
12	M/3.0	Right thigh	Hypertrichosis	No	Negative	Yes	Yes	**–**	**–**	Chinese patent medicine	Stable
13	M/5.0	Right buttock, right thigh	None	No	Negative	Yes	Yes	**–**	**–**	**–**	Stable
14	F/6.0	Right buttock, right thigh	Subcutaneous nodules	No	Negative	Yes	Yes	–	–	Chinese patent medicine	Stable
15	M/5.0	Left shoulder	None	No	Negative	Yes	Yes	–	Yes	Chinese patent medicine	Stable
16	F/8.0	Left thigh	None	No	Negative	Yes	Yes	Yes	–	Chinese patent medicine	Stable

^a^In addition to skin sclerosis, the patients had the following symptoms; MMF, mycophenolate mofetil; MTX, methotrexate; –, not mentioned. PUMCH, Peking Union Medical College Hospital

**Fig. 2:**
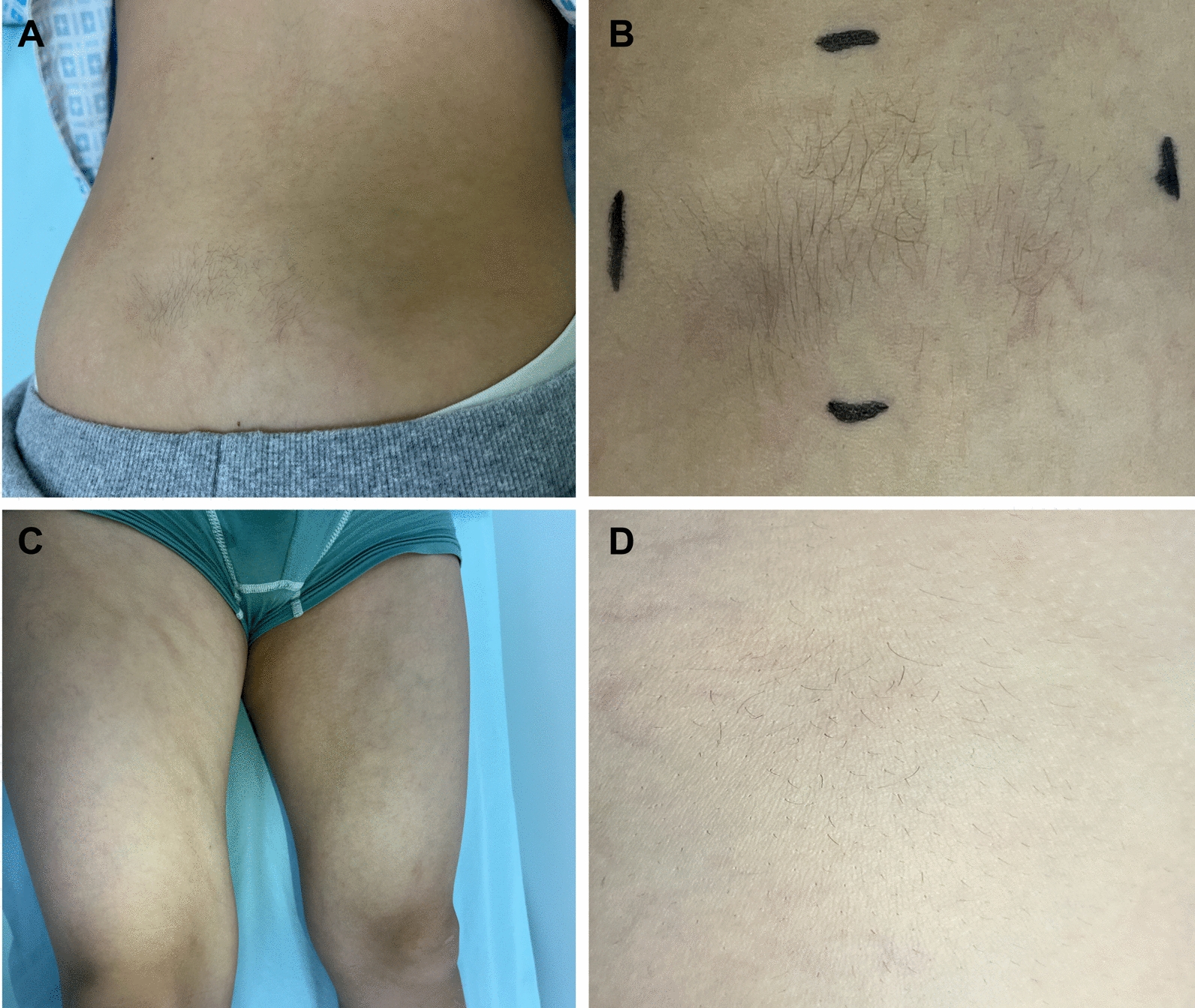
Clinical characteristics of stiff skin syndrome. **A**, **B** A boy diagnosed with segmental stiff skin syndrome presenting with sclerosis and hypertrichosis of the lumbosacral area.** C**, **D** A boy diagnosed with segmental stiff skin syndrome showing a cobblestone appearance and hypertrichosis on the right thigh

All patients presented with varying degrees of skin hardening, associated with hypertrichosis (5, 31.3%), hyperpigmentation (2, 12.5%) and a cobblestone appearance (5, 31.3%) (Fig. 2). The skin hardening predominantly affected the lower limbs in SSS patients (13, 81.3%), and the left side was involved in the majority of patients with segmental SSS (8, 57.1%). In contrast, skin hardening was bilateral in patients with widespread SSS.

**Fig. 3:**
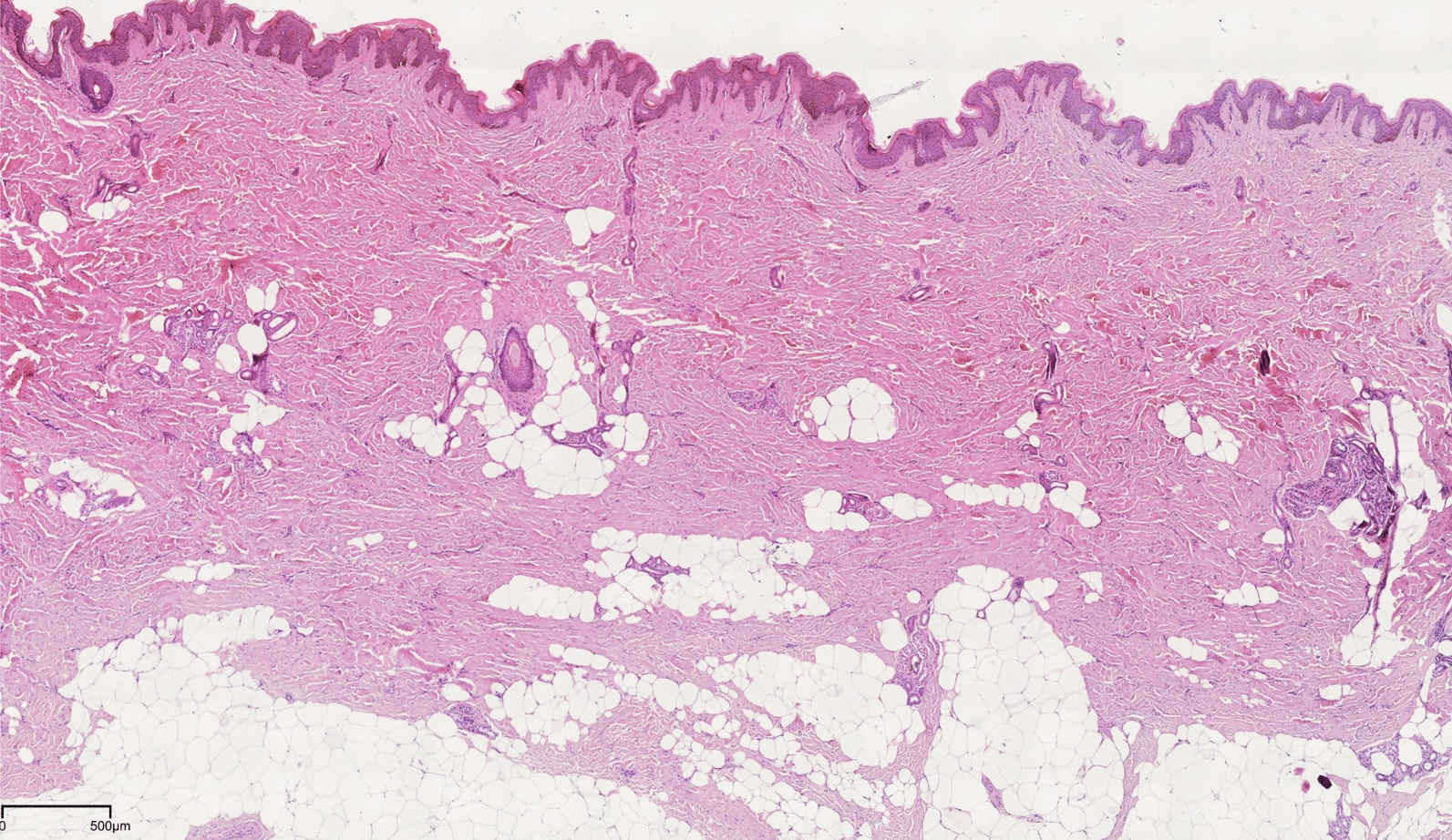
Histopathology of stiff skin syndrome. Epidermal hyperkeratosis, mild papillomatous hyperplasia, hyperpigmentation of the basal layer, increased deposition of collagen fibers and fibroblast hyperplasia. Dermal entrapment of adipocytes was observed. There were no obvious abnormalities in the subcutaneous fat tissue (Hematoxylin–eosin stain; original magnification × 40)

Laboratory investigations revealed normal inflammatory markers (C-reactive protein and/or erythrocyte sedimentation rate), immunoglobulins and complement for all patients. Two patients exhibited low titers of autoantibodies: one case with weak positive antibodies against proliferating cell nuclear antigen and the other one against ribonucleoprotein. Notably, all these autoantibody levels resolved within a short period. Skin histopathology demonstrated thickening of the dermis (16, 100%), adipocyte entrapment by thickened and horizontally oriented collagen bundles (8/9, 88.9%), and absence of infiltration by inflammatory cells. Alcian blue staining was positive in 5 patients (5/5, 100.0%) (Fig. 3).

Regarding treatment, all patients received physical therapy. Ten patients (62.5%) were treated with Chinese patent medicine, resulting in disease stability, with 1 patient lost to follow-up. Following ineffective physical therapy, two patients showed symptom relief after adding mycophenolate mofetil (MMF), while another 2 patients did not exhibit significant improvement after initiating Jak inhibitors.

During a follow-up period of 6.5 ± 3.5 years, only 3 patients developed joint contractures (1 in the right elbow joint and 2 in the left hip joint). None of the segmental SSS patients progressed to widespread SSS, and there were no reports of respiratory, gastrointestinal, or hematologic involvement, nor complaints of Raynaud’s phenomenon or fingertip ulcers.

### Literature review

**Fig. 4:**
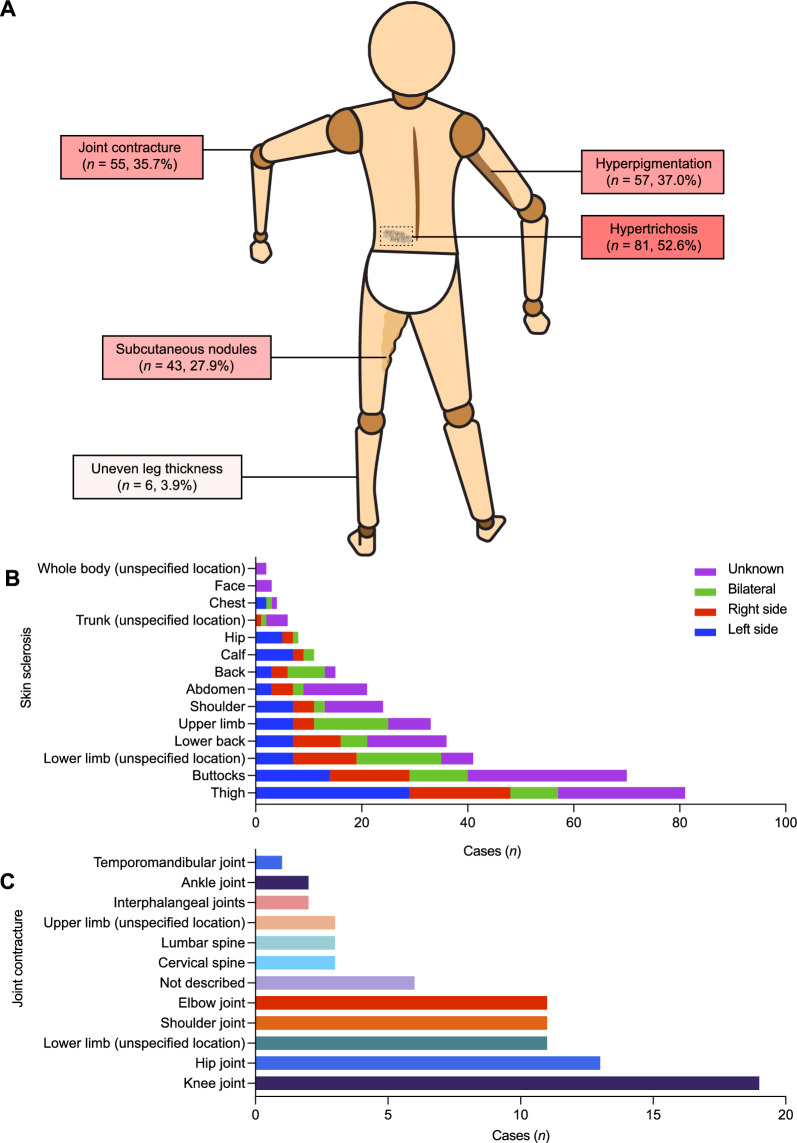
Clinical manifestations of patients with stiff skin syndrome. **A** patient with stiff skin represented like a “puppet”, the box show the different clinical manifestations, the number of cases and proportions; **B** bar chart showing the location and corresponding number of cases of skin sclerosis; **C** chart showing the distribution of joint contractures

A total of 56 articles (138 cases) on childhood-onset SSS from January 2000 to January 2024 were identified in PubMed, Wanfang data, and CNKI databases, including 21 Chinese articles (45 cases) [5–25] and 34 English articles (93 cases) [3, 26–53]. The clinical manifestations of the patients in the literature and our hospital (*n* = 154) were summarized (Fig. 4A, Table 2).

**Table 2:** Clinical manifestations of stiff skin syndrome patients

Variables	All patients (*n*, %)	Segmental SSS (*n*, %)	Widespread SSS (*n*, %)
Total number(*n*)	154(100)	97(63.0)	36(23.4)
Sex (*n* = 154)			
Male	65 (42.2)	42 (27.3)	16 (10.4)
Female	89 (57.8)	55 (35.8)	20 (13.0)
Age of onset (years)	2.0 (0.5, 4.8)	2.0 (0.5, 5.0)	1.5 (0.3, 4.0)
Age of diagnosis (years)	8.5 (5.0, 13.0)	8.0 (5.0, 12.0)	9.5 (6.0, 17.0)
Diagnostic interval (years)	5.5 (2.0, 10.0)	5.0 (1.6, 9.0)	8.0 (3.5, 14.0)
Family history(*n* = 154)	19 (12.4)	9 (5.8)	8 (5.2)
Population distribution(*n* = 154)			
China	98 (63.6)	62 (40.3)	15 (9.7)
America	15 (9.7)	10 (6.5)	5 (3.2)
Canada	12 (7.8)	10 (6.5)	2 (1.3)
Brazil	7 (4.5)	4 (2.6)	3 (1.9)
Spain	6 (3.9)	6 (3.9)	0
Poland	5 (3.2)	2 (1.3)	3 (1.9)
Britain	3 (1.9)	0	3 (1.9)
Italy	2 (1.3)	1 (0.6)	1 (0.6)
Portugal	1 (0.6)	0	1 (0.6)
Turkey	1 (0.6)	0	1 (0.6)
Lebanon	1 (0.6)	0	1 (0.6)
Unknown	3 (1.9)	2 (1.3)	1 (0.6)
Genetic test (*n* = 20)			
*FBN1*	6 (30.0)	0	6 (30.0)
*IL17C*	1 (5.0)	1 (5.0)	0
Misdiagnosi*s* (*n* = 49)			
Localized scleroderma	37 (75.5)	19 (38.8)	13 (26.5)
Systemic sclerosis	3 (6.1)	0	3 (6.1)
Eosinophilic fasciitis	3 (6.1)	3 (6.1)	0
Connective tissue nevus	5 (10.2)	5 (10.2)	0
Eczema	2 (4.1)	2 (4.1)	0
Superficial lipomatous nevus	1 (2.0)	1(2.0)	0
Seronegative arthritis	1 (2.0)	0	1 (2.0)
Gluteal muscle contracture	1 (2.0)	0	1 (2.0)
Buschke scleredema	2 (4.1)	1(2.0)	1 (2.0)
Dermatomyositis	1 (2.0)	0	1 (2.0)
Telangiectasia nevus	1 (2.0)	0	1(2.0)
Skin manifestations (*n* = 154)			
Scleroderma	154 (100.0)	97 (63.0)	36 (23.4)
Hyperpigmentation	57 (37.0)	38 (24.7)	11 (7.1)
Hypertrichosis	81 (52.6)	39 (25.3)	21 (13.6)
Subcutaneous nodules	43 (27.9)	19 (12.3)	20 (13.0)
Joint contracture	55 (35.7)	24(15.6)	29(18.8)
Organ involvement (*n* = 154)			
Muscular system	3 (1.9)	1 (0.6)	2 (1.3)
Respiratory system	2 (1.3)	0	2 (1.3)
Digestive system	3 (1.9)	1 (0.6)	2 (1.3)
Circulatory system	1 (0.6)	0	1 (0.6)
Nervous system	2 (1.3)	0	2 (1.3)
Eyes	3 (1.9)	0	3 (1.9)

Continuous variables with non-normal distribution were represented by *M* (*Q*_1_, *Q*_3_). Categorical variables were expressed as *n* (%)

Among the SSS patients with childhood onset, 89 (57.8%) were female. Apart from the 21 cases reported by Wang et al. [13], disease subtypes or detailed descriptions of cutaneous manifestations were provided for all other patients. Among them, there were 97 patients (63.0%) with segmental SSS and 36 patients (23.4%) with widespread SSS. The average age of onset was 2.0 (0.5, 4.8) years, with the age of diagnosis being 8.5(5.0, 13.0) years, and an interval between onset and diagnosis of 5.5 (2.0, 10.0) years. Positive family history (skin sclerosis in 1 st degree family members) was noted in 19 patients (12.3%). Two cases had consanguineous parents, but family history and genetic testing results were negative. Gene sequencing was performed in 20 patients, among whom 7 patients (35.0%) showed positive results (6 patients carrying fibrillin 1 *(FBN1)* gene variants (3 patients carried *p*.Trp1570Cys, 2 patients carried *p*.Trp1570Arg, and 1 patient carried an *FBN1* variant without further specification), and 1 patient with interleukin-17C (*IL17C*) variant (c.532_543dupTTCCACACCGAG, *p*.Phe78_Glu181dup)). Initially, forty-nine patients (31.8%) were misdiagnosed with other diseases, including localized scleroderma (37, 75.5%), connective tissue nevi (5, 10.2%), systemic sclerosis (3, 6.1%), and eosinophilic fasciitis (3, 6.1%). Five patients were misdiagnosed with more than one conditions, including scleroderma and eosinophilic fasciitis, among others.

Clinically, all patients presented with skin sclerosis primarily affecting the thigh (81, 52.6%) and buttock (70, 45.5%) (Fig. 4B). Hyperpigmentation was observed in 57 patients (37.0%), hypertrichosis on skin lesions in 81 patients (52.6%), and subcutaneous nodules in 43 patients (27.9%) (Fig. 4A). Joint mobility restrictions secondary to skin hardening were reported in 55 patients (35.7%), mainly involving the knees (19, 12.3%), hips (13, 8.4%), elbows (11, 7.1%), and shoulder joints (11, 7.1%) (Fig. 4C). However, none exhibited joint redness or swelling.

Six patients (3.9%) had uneven circumference of legs due to growth restrictions caused by skin stiffness. Additionally, five patients (3.2%) had short stature while 3 (1.9%) had low body weight. Two patients displayed chronic compartment syndrome in their lower extremities which improved after surgical intervention. One patient had myogenic lesions, suggested by electromyography without muscle weakness. Three patients from a family consisting of a mother and her two daughters had extraocular muscle paralysis leading to restricted orbital movement. The majority of patients with SSS showed no visceral involvement. Among those with systemic manifestations, mild organ involvement was observed, primarily affecting the respiratory (2, 1.3%), gastrointestinal (3, 1.9%), and cardiovascular systems (1, 0.6%).

Patients with SSS were prone to joint contractures (Table 3). In this cohort of 128 SSS patients with detailed joint involvement documentation, widespread SSS were more susceptible to joint contractures compared with segmental SSS (*P* < 0.001). Moreover, prolonged intervals from disease onset to diagnosis (*P* = 0.030) and upper limb skin sclerosis are associated with increased risk of joint contractures (*P* < 0.001). There were no statistically significant differences between patients with and without joint contractures regarding sex, age, family history, or characteristics of skin lesions.

**Table 3:** Differences in clinical manifestations of patients with joint contracture

Variables	Joint contracture (*n* = 51)	No joint contracture (*n* = 77)	*P*
Types (segmental/widespread)	24/27	69/8	**< 0.001**^a^
Sex (male/female)	18/33	36/41	0.199^a^
Age of onset (years)	1.8 (0.7,5.0)	2.0 (0.4, 4.0)	0.477^b^
Diagnostic interval(years)	11.0 (6.0,15.3)	4.7 (1.8, 8.5)	**0.030**^b^
Positive family history (*n* = 17)	8 (15.7)	9 (11.7)	0.514^a^
Hyperpigmentation (*n* = 49)	16 (31.4)	33 (42.9)	0.191^a^
Hypertrichosis (*n* = 57)	24 (47.1)	33 (42.9)	0.640^a^
Subcutaneous nodules (*n* = 26)	11 (21.6)	15 (19.5)	0.774^a^
Lower limb(s) skin sclerosis (*n* = 104)	41 (80.4)	63 (81.8)	0.840^a^
Upper limb(s) skin sclerosis (*n* = 26)	20(39.2)	6(7.8)	**< 0.001**^**a**^

Bold values indicated statistical significance (*P* < 0.05)

^a^Compared by Chi-square test

^b^Compared by non-parametric test

Continuous variables with non-normal distribution were represented by *M* (*Q*_1_, *Q*_3_). Categorical variables were expressed as* n* (%)

Laboratory investigations revealed normal inflammatory markers (C-reactive protein and/or erythrocyte sedimentation rate) in all patients, with only 4 patients (4/73, 5.5%) had positive autoantibodies. Three patients (3/59, 5.1%) had immunoglobulin abnormalities (3/59, 5.1%), and there were 2 patients (2/56, 3.6%) with transient low complement. In skin biopsy, only 20 patients had biopsies reaching the fascial layer, of which 10 patients (50.0%) showed thickening of the fascia. Most patients presented with fibroblast proliferation (51/82, 62.2%), dermal collagen fiber bundles (108/112, 96.4%), focal entrapment of mature adipocytes in the sclerotic collagen (55/66, 83.3%), Alcian blue staining suggesting varying degrees of mucin deposition (49/78, 62.8%). However, lymphocyte infiltration was relatively rare (12/95, 12.6%).

After a definitive diagnosis of SSS, all patients received physical therapy. Some patients also received traditional Chinese medicine, with varying degrees of reported efficacy. Some patients additionally received MMF, methotrexate (MTX), losartan (LST), etc., but the reported effectiveness also varied (Table 4). Two patients with SSS in our hospital were treated with MMF because of ineffective physical therapy. Combined with previous literature, a total of 10 patients were treated with MMF, among whom 8 had segmental SSS, with 7 patients showing a positive response. In the treatment of 2 cases of widespread SSS and 5 cases of segmental SSS with LST (Table 4). The initial dosage of LST therapy was 12.5 mg/day or 0.7 mg/kg/day, gradually increasing to an effective dose of 12.5–50 mg/day. A minimum treatment duration of 1 month was sufficient to observe therapeutic effects. One patient experienced alleviation of skin lesions with LST treatment but discontinued due to somnolence and nausea. Another patient discontinued the treatment due to the ineffectiveness of LST in alleviating skin sclerosis in the lower extremities.

**Table 4:** Medical treatment in patients with stiff skin syndrome

Patients	Sex	Types	Treatment	Treatment duration	Adverse events	Therapeutic response
1[52]	M	widespread	MMF	6–12 months	None	None
			Secukinumab	> 12 months	None	None
2[52]	M	segmental	Calcineurin inhibitor	< 6 months	None	None
3[52]	M	widespread	MTX	1 year	None	None
4[52]	M	segmental	MTX + prednisone	6–12 months	None	None
5[52]	F	segmental	Topical steroid	Not described	None	None
6[30]	M	widespread	Methylprednisolone + MTX	Methylprednisolone (20 mg/kg) for 3 months along with MTX (25 mg/week) for 1 year	None	Yes
7[49]^a^	F	segmental	Deflazacort	25 mg/day for 1 month	None	Mild-moderate
			MTX	7.5 mg/week for 7 months	None	None
			LST	12.5 mg/day for 10 months	None	Mild
				18.75 mg/day for 5 months	None	Mild
8[49]^a^	F	segmental	MTX	15 mg/week for 16 months	Gastrointestinal symptoms	None
			LST	12.5 mg/day for 1 month	Somnolence, dizziness	Mild–moderate
9[49]^a^	F	segmental	MMF	1 g/12 h for 24 months	None	Moderate
10[49]^a^	F	segmental	MMF	1 g/12 h for 9 months	None	Mild
			LST	12.5 mg/day for 1 month	None	Stable
11[49]^a^	M	segmental	LST	12.5 mg/day for 1 month	None	None
				25 mg/day for 8 months	None	Mild
			MMF	0.75 g/12 h for 5 months	None	Subtle new lesions on left lower back at most recent follow-up
12[51]	F	segmental	MTX/MMF	Not described	Nausea, fatigue	None
			Secukinumab	150 mg every 4 weeks for 4 months	None	Skin induration and joint limitation remained stable
13[48]	M	segmental	LST	0.7 mg/kg/day and titrated to 50 mg/day	None	Mild
14[46]	F	segmental	MTX	25 mg/week for 3 months	None	None
			MMF	3 g/day for 6 months	None	Subjective and objective softening of lesional skin
15[46]	F	segmental	MMF	2 g/day for 3 months	None	subjective and objective softening of lesional skin
16[41]	M	widespread	LST	Not described	None	None
17[2]	M	segmental	MTX	10 mg/week for 1 year	None	Condition remaining clinically stable
18[29]	M	segmental	MTX	7.5 mg/week and then increased to 10 mg weekly	None	Disease stability
19[47]	F	widespread	LST	12.5 mg/day for 12 months	None	Skin induration and joint limitation remained stable
			MMF	0.18 g/12 h and then adjusted to 0.18 and 0.36 g/day alternating daily for 12 months	None	Skin induration and joint limitation remained stable
20^b^	M	segmental	MTX	10 mg/week for 0.5 months	None	Softening of lesional skin
			MMF	25 mg/kg for 12 months	None	Softening of lesional skin
21^b^	F	segmental	MMF	30 mg/kg for 12 months	None	Softening of lesional skin, no progression of sclerosis
22^b^	M	segmental	Tofacitinib	5 mg/day 2 months	None	None
23^b^	F	segmental	MTX	10 mg/week for 1 month	None	None
			Baritinib	2 mg/day for 2 months	None	None

^a^In reference 49, high-frequency ultrasonography failed to reflect the clinical improvements noted in these patients

^b^These four patients were derived from the PUMCH cohort

F, female; M, male; MMF, mycophenolate mofetil; MTX, methotrexate; LST, losartan

Among the 78 patients (50.6%) with follow-up ranging from 1 month to 17 years, 45 patients (57.7%) remained stable, and 8 patients (10.3%) exhibited some reduction in skin sclerosis, yet their skin did not revert to a normal appearance. One patient experienced gradual softening of affected skin lesions during pregnancy. Eighteen patients (23.1%) had an extending skin lesions or worsening skin hardening. All patients showed no involvement of multiple visceral organs.

## Discussion

Stiff skin syndrome is a rare, autosomal dominant, cutaneous disorder that presents at birth or during childhood. Our study thoroughly summarized the clinical features, treatment and prognosis of childhood-onset SSS patients, combining literature and PUMCH cohort. SSS typically present either at birth or before the age of 6, predominantly as the segmental type. Segmental SSS (mean onset age 4.1 years) tends to occur later than widespread SSS (mean onset age 1.6 years) [3]. In our study, the median age of onset for SSS patients was 2 years, consistent with findings from previous research.

SSS frequently affects fascia-rich areas such as the buttocks and thighs, clinically presenting with a “board-like” appearance. Some patients may also exhibit hyperpigmentation, hypertrichosis, subcutaneous nodules at the affected skin. All patients diagnosed with SSS have skin lesions; however, in our study, these skin lesions predominantly involved the lower limbs. All cases of segmental SSS did not progress to bilateral involvement, consistent with previous literature [3].

Approximately 97% of SSS patients may experience joint mobility restriction secondary to skin sclerosis [37]. Reviewing literature from 2000 to 2024 and cases seen at our institution, only 55 patients (35.7%) presented with joint mobility restriction secondary to skin sclerosis, which differs from previous reports, possibly attributed to global economic development, enhanced healthcare awareness, and early diagnosis and treatment of the disease. This study also found that the diagnostic interval was longer for patients with joint contractures compared to those without. Therefore, it is recommended that pediatricians pay attention to the skin examination during clinical diagnosis and treatment, aim for early diagnosis and early intervention, and actively promote rehabilitation exercises to prevent the occurrence of joint contractures.

Generally, SSS is a diagnosis of exclusion, with a distinctive clinical presentation but no pathognomonic laboratory or pathological findings [43, 44]. Typically, patients do not exhibit skeletal muscle or visceral organ involvement, nor do they have immunological abnormalities. Retrospective analysis showed that SSS patients in PUMCH did not have internal organ involvement, which was consistent with previous studies [53]. Despite the presence of autoantibody positivity in two patients from our center’s cohort of SSS patients, these were either low-titer or transient, and disappeared within a short period.

The histopathological features of SSS include thickened collagen bundles, increased connective tissue mucin, horizontal orientation of thickened collagen bundles with adipocyte entrapment and lack of inflammation [3]. SSS exhibits a spectrum of histopathologic findings, with interstitial mucin representing early disease and the deep dermal sclerosis signifying a later stage [30]. In our research, all patients had skin hardening and some patients had hyperpigmentation, hypertrichosis, consistent with the clinical presentation of SSS. After excluding systemic sclerosis [54], localized scleroderma [55], and eosinophilic fasciitis [56] through systematic evaluation and biopsy, all patients could be definitively diagnosed with SSS.

Physical therapy serves as the cornerstone of comprehensive management for patients with SSS [30]. Approximately 30% of SSS patients have been reported to be associated with genetic factors [35]. Literatures reported that *FBN1* gene (15q21.1) variants may be responsible for the autosomal dominant form of widespread SSS [57]. However, segmental SSS patients are not monogenic in pathogenesis [3, 30, 47]. Among the patients in literature review, six had *FBN1* gene variants [38, 41, 52]. Variants in *FBN1* may cause increased activation of transforming growth factor-β (TGF-β) signaling, leading to increased TGF-β concentrations, fibroblast proliferation and the development of a profibrotic phenotype [42, 57, 58]. Abnormal TGF-β signaling pathways and fibrosis were common features of the classic SSS, which opened a way to the potential drugs regulating the TGF-β signaling pathway and reducing fibrosis [46, 48]. As an angiotensin II receptor blocker, LST can reduce thrombospondin-1 levels, suppressing the activation of TGF-β and alleviating fibrogenesis [42, 47, 49]. Literature review identified seven patients treated with LST, among whom only one case showed no improvement in SSS related symptoms. Thus, LST may be a potential therapeutic option for SSS.

MMF may reduce fibroblast proliferation by inhibiting guanosine nucleoside formation and decreasing the concentration of TGF-β in peripheral blood, which support its use in the treatment of SSS in previous studies. Currently, it is widely used in the treatment of SSc [59]. However, reports on the use of MMF for SSS were relatively scarce, mostly consisting of case studies. Combining literature data with our cohort, ten patients received MMF treatment, with 7 showing symptom improvement [46, 47, 49], suggesting that MMF could potentially serve as an effective therapeutic option for patients with rapidly progressive SSS [47]. However, the efficacy still needs to be confirmed by larger, multicenter studies or collaborative efforts to gather sufficient evidence.

As evidenced in some literature, IL-17 has been associated with the pathogenesis of SSS [51]. Secukinumab, which exhibits cross-reactivity and blocks both IL-17A and IL-17C, has been documented in a single patient to soften the skin and improve joint mobility restrictions [51], while no improvement was observed in another case [52]. Furthermore, a case study reported that a 56-year-old male patient with SSS following myeloma experienced improvements in skin lesions and joint mobility after hematopoietic stem cell transplantation [60]. However, considering the relatively favorable prognosis of SSS, medical treatment in SSS should be carefully performed after weighing the pros and cons, with close monitoring and efficacy evaluation [47].

We summarized the clinical characteristics of patients with SSS from PUMCH and prior literatures, all of whom were definitively diagnosed with SSS based on clinical and pathological findings. However, this study also has some limitations. Some of the literature did not provide a detailed description of the pathological findings, rendering the summary of pathological results relatively challenging. Another limitation of this study was its single-center nature. While we have compiled a summary of all SSS cases by reviewing relevant literature, the number of cases remains relatively low, insufficient to draw definitive conclusions on treatment protocols. Therefore, future multi-center registry studies encompassing a global scope are warranted.

## Conclusions

SSS is a rare dermatological condition prone to misdiagnosis and missed diagnosis in its early stages. For patients with early-onset skin sclerosis, severe joint contractures, and inadequate response to medication, biopsy and genetic testing should be considered to differentiate SSS from mimics. Early recognition is crucial to avoid prolonged use of ineffective therapeutic modalities. Physical therapy is highly recommended to prevent joint contractures. Treatment options are scarce and aim to regulate the expression of TGF-β and, consequently, inhibit the profibrotic pathogenesis. MMF, LST, Chinese patent medicine and other drugs that act on the TGF-β pathway may delay the progression of the disease but cannot reverse the skin lesions.

## Data Availability

The datasets are available from the authors upon reasonable request and with permission of the Institutional Review Board of Peking Union Medical College Hospital.

## Abbreviations

CNKI: China national knowledge infrastructure
IL-17: Interleukin-17
LST: Losartan
MMF: Mycophenolate mofetil
MTX: Methotrexate
PUMCH: Peking union medical college hospital
SSS: Stiff skin syndrome
TGF-β: Transforming growth factor-β
